# Evaluation of hepatic infantile hemangiomas: the potential of contrast-enhanced ultrasound

**DOI:** 10.3389/fcvm.2026.1886544

**Published:** 2026-09-08

**Authors:** Gabriella Cericola, Lennart Gunst, Haroun Bel Hadj Jrad, Jörg Sahlmann, Alexander Puzik, Christian Voll, Alexandros Rahn, Doris Franke, Friedrich G. Kapp

**Affiliations:** 1 Department of Pediatric Hematology, Oncology and Stem Cell Transplantation, Faculty of Medicine, Children's Hospital, Medical Center, University of Freiburg, VASCERN VASCA European Reference Centre, Freiburg, Germany; 2 Department of Pediatric Sonography, Faculty of Medicine, Children's Hospital, Medical Center, University of Freiburg, Freiburg, Germany; 3 Department of Radiology, Faculty of Medicine, Medical Center, University of Freiburg, Freiburg, Germany; 4 Institute for Medical Biometry and Statistics, Faculty of Medicine, Medical Center, University of Freiburg, Freiburg, Germany; 5 Department of Pediatric Neonatology, Faculty of Medicine, Children's Hospital, Medical Center Dritter Orden-Paussau, TU Munich, Passau, Germany; 6 Department of Pediatric Pulmonology, Allergology and Neonatology, Hannover Medical School, Hannover, Germany; 7 Department of Pediatric Kidney, Liver and Metabolic Diseases, Hannover Medical School, Hannover, Germany

**Keywords:** contrast-enhanced ultrasound, diagnostic comparison, hepatic infantile hemangioma, magnetic resonance imaging, pediatric imaging

## Abstract

**Objectives:**

To evaluate laboratory parameters and to compare diagnostic findings of contrast-enhanced ultrasound (CEUS) and magnetic resonance imaging (MRI) in a cohort of patients with hepatic infantile hemangiomas (IH).

**Materials and Methods:**

A retrospective multicenter analysis was conducted to identify infants diagnosed with hepatic IH who underwent CEUS, MRI, or both between 2016 and 2024. Lesion size and count were compared in patients who underwent both imaging modalities (6 patients). Data from 24 patients were included in the analysis; in 18 of them, only a single diagnostic imaging examination was performed.

**Results:**

Laboratory parameters were mostly unremarkable, only Pro-Brain Natriuretic Peptide (proBNP) was elevated in a subset of patients. Of 24 patients, 15 underwent only CEUS, 3 underwent only MRI, and 6 received both imaging modalities. CEUS findings were comparable to MRI regarding lesion size and number. Treatment decisions were predominantly based on CEUS findings.

**Conclusion:**

Although laboratory abnormalities were uncommon, targeted assessment of proBNP and thyroid function may be clinically relevant in infants with extensive hepatic involvement. Both imaging modalities detected hepatic IH. CEUS demonstrated diagnostic findings largely consistent with MRI in hepatic hemangioma evaluation, suggesting its potential as a complementary tool without compromising disease severity assessment. CEUS advantages include real-time imaging and avoidance of sedation or anesthesia in young patients. These findings support the potential role of CEUS as a viable, readily available, and resource-efficient alternative to MRI for the evaluation of hepatic IH, enabling optimization of diagnostic and treatment protocols.

## Introduction

Infantile hemangiomas (IH) are classified as benign vascular tumors according to ISSVA classification (1) and affect 4%–5% of newborns, with a higher prevalence in females and preterm infants. They typically emerge shortly after birth, undergo rapid proliferation during the first 4–6 months of life and gradually involute during early childhood, with residual findings persisting in some cases (2). Although the etiology remains unclear, a multifactorial origin is assumed. Recent findings implicate the SOX18 and the mevalonate pathway as potential targets of R(+)-propranolol, suggesting molecular mechanisms that may underlie both hemangioma formation and regression (3, 4).

Hepatic hemangiomas in infancy are subclassified into focal, multifocal, and diffuse types, each with distinct imaging features and clinical implications (5). Congenital hemangiomas are typically focal, solitary and asymptomatic. By contrast, hepatic IH are typically multifocal or diffuse and frequently associated with cutaneous hemangiomas. Multifocal lesions consist of multiple nodules interspersed with normal liver tissue, while diffuse hemangiomas replace large hepatic areas. While hepatic IH represents a rare subset of IH, they are important to identify, since extensive or diffuse hepatic disease may be associated with high-output cardiac failure, pulmonary hypertension, abdominal compartment syndrome, and consumptive hypothyroidism—complications that have become rare with propranolol therapy (6, 7). Despite propranolol being the current treatment standard, diagnostic approaches remain heterogeneous. Hepatic involvement should be ruled out in infants with ≥5 cutaneous hemangiomas (8, 9). Conventional ultrasound (US) is the main imaging modality for screening for hepatic involvement, but follow-up in patients with hepatic lesions identified in ultrasound remains highly individualized. While the rate of suspicion for hepatic IH may be high for patients with multiple cutaneous IH and hepatic lesions, the latter may represent a multitude of benign and malignant differential diagnoses (10, 11), making a reliable diagnostic tool desirable.

Magnetic resonance imaging (MRI) with and without contrast is currently the reference modality for hepatic lesion characterization. In infants, MRI may require sedation or anesthesia depending on age, motion, examination duration; however, sedation is not always mandatory, e.g., at institutions with established feed-and-wrap techniques (12). MRI may also involve gadolinium-based contrast agents when contrast-enhanced characterization is required.

Contrast-enhanced ultrasound (CEUS) with sulfur hexafluoride microbubbles (Lumason/SonoVue, Bracco Diagnostics) is a promising alternative. CEUS provides real-time dynamic evaluation of vascularization, detecting microvessels that are invisible on Doppler imaging and can be performed without sedation. Although approved in Europe for adult liver imaging, and in the U.S. for pediatric use, its pediatric indication remains off-label in Europe. Contraindications include right-to-left cardiac shunts and severe pulmonary hypertension (13).

CEUS is thought to offer high diagnostic accuracy, excellent safety, and potential cost advantages for focal liver lesions compared to MRI (14, 15). However, larger pediatric cohorts that assess different imaging modalities for the evaluation of suspected hepatic IH are lacking.

The present multicenter retrospective study aimed to describe real-world clinical and laboratory data from infants with hepatic IH, with a specific focus on the diagnostic approaches to suspected liver IH using CEUS and MRI. By evaluating these techniques, we aim to optimize the diagnostic approach, potentially reducing reliance on more resource-intensive procedures and accelerating diagnosis while maintaining or improving accuracy.

## Materials and methods

### Patient selection

This multicenter retrospective study reviewed CEUS and MRI examinations of patients with hepatic IH. Written informed consent was obtained from parents or legal guardians, and the study was approved by the institutional review boards of the University Medical Center Freiburg (21-1200) and Hannover (10527_BO_K_2022). Data collection spanned from October 1, 2016, to October 2024.

Conventional ultrasound was performed as a screening method in patients with multifocal cutaneous hemangiomas; the additional imaging procedures were then selected based on the patient's clinical presentation, local institutional practice, and the diagnostic requirements of the individual case.

**Figure 1:**
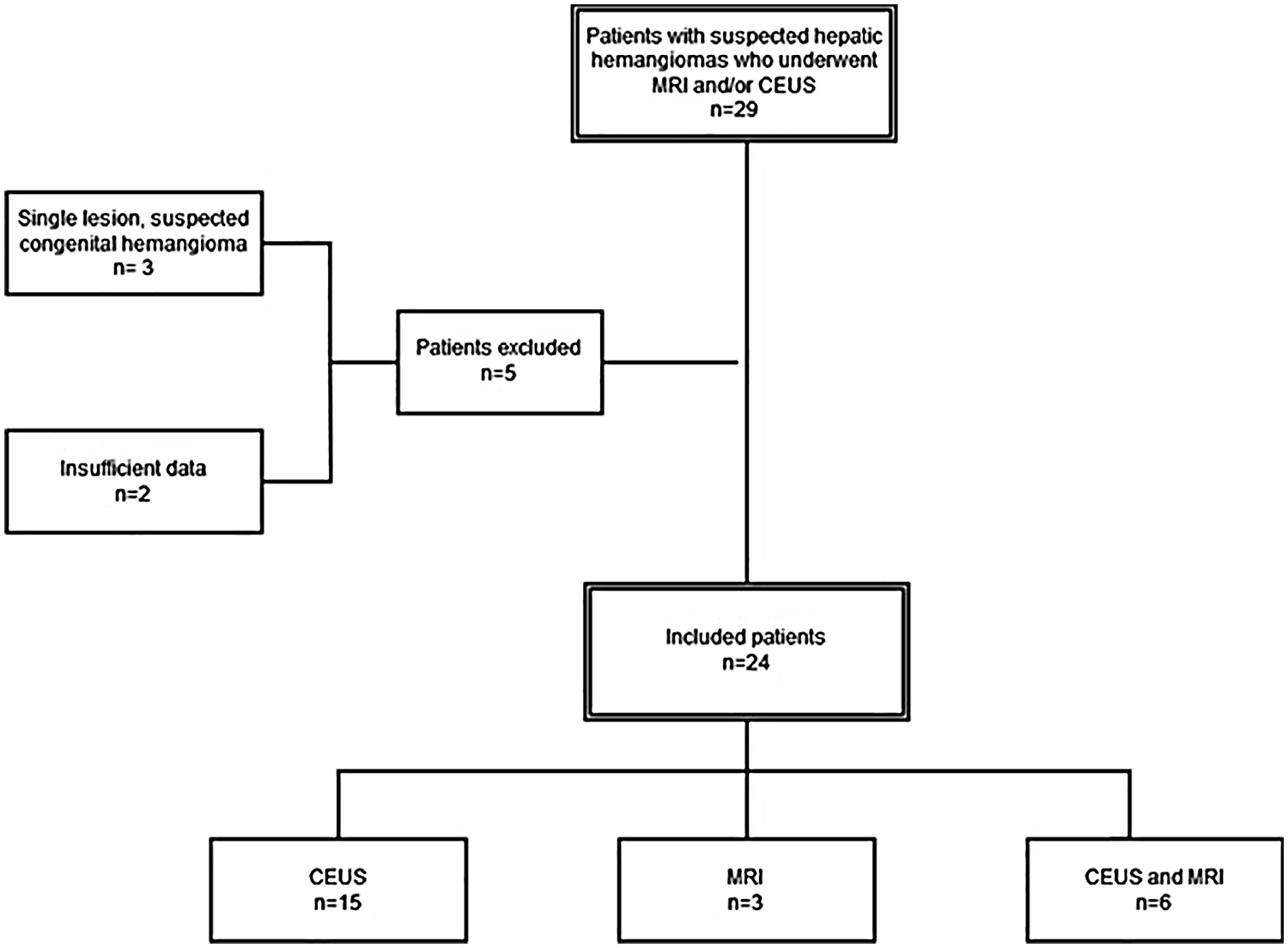
Study population. This flow diagram depicts the patient selection process for our study on infantile hepatic hemangiomas.

A total of 29 patients were identified; five were excluded (three due to solitary congenital hemangiomas and two for incomplete data), resulting in 24 eligible infants (Figure 1). All imaging studies for lesion characterization were performed prior to initiation of propranolol therapy. CEUS and MRI examinations used for direct comparison were conducted in close temporal proximity (maximum interval 10 days), except for patient P6, in whom a 38-day interval occurred due to prematurity. In this case, CEUS suggested hepatic IH, while MRI and treatment were deferred under close sonographic monitoring until the estimated due date; no lesion growth was observed during this period.

Collected data included lesion number, size and imaging features, as well as clinical information (age, sex, prematurity, and laboratory results, treatment and outcomes).

Treatment outcome was classified according to the last available follow-up imaging. Complete response was defined as the absence of detectable hepatic lesions. Regression was defined as a reduction in lesion size, number, and/or vascularity, with residual hepatic lesions still detectable at the last follow-up examination.

### Contrast-enhanced ultrasound (CEUS)

CEUS exams were performed using multiple ultrasound systems (Zonare ZS3 Mindray, GE Logiq E10, E9, S8, and Toshiba Aplio XG). The contrast agent SonoVue was used with a dose of 0.03 mL/kg body weight in all patients, followed by a 5–10 mL saline flush; contrast dynamics were comparable across examinations. Imaging included a 20-second dynamic sequence post-injection and static captures at one-minute intervals for 5–6 min. IH diagnosis was based on typical enhancement patterns: early peripheral nodular enhancement with centripetal fill-in during the arterial phase, becoming homogeneous in later phases (Supplementary Video 1). An experienced pediatric sonographer interpreted all CEUS exams. No CEUS-related adverse events, allergic reactions, or contrast-agent-related complications were observed during the study period. In our cohort, none of the patients required sedation for CEUS examinations.

### Magnetic resonance imaging examination

Four patients underwent contrast-enhanced MRI using T1-weighted Turbo Spin Echo and short tau inversion recovery (STIR) sequences with ProHance. Hemangiomas showed peripheral nodular enhancement with delayed central filling, hyperintensity on T2-weighted, and no signal on pre-contrast T1 sequences. The remaining five patients underwent non-contrast MRI on 1.5 T or 3.0 T scanners, with standard pediatric protocols, including T1- and fat-suppressed T2-weighted imaging, diffusion-weighted imaging (b-values of 50, 400, and 800 s/mm²) and apparent diffusion coefficient (ADC) mapping. In-phase/out-of-phase sequences were used to assess hepatic fat content. An experienced pediatric radiologist evaluated all MRI images, documenting abnormal enhancement and notable findings in T1 and STIR sequences. In our cohort, all the patients received sedation for MRI examinations.

### Statistical analysis

Agreement between CEUS and MRI lesion size measurements was explored using scatter plots and Spearman's rank correlation coefficient (*ρ*), comparing CEUS (*x*-axis) and MRI (*y*-axis), focusing on the largest lesion in a defined anatomical region to compare matched lesions. Given the limited number of patients who underwent both CEUS and MRI (*n* = 6), analyses were considered exploratory. No formal regression testing for normality or multicollinearity was applied.

## Results

### Patient characteristics

Patient characteristics are depicted in Table 1 and Supplementary Table S1. This multicenter study analyzed 24 infants diagnosed with hepatic IH. 23 patients had multifocal hepatic hemangiomas, while one (P20) presented with diffuse involvement. There was a female predominance with 18 females (75%). Age at diagnosis ranged from birth to eight months (median: 3 months) (Table 1 and Supplementary Table S1).

**Table 1:** Patient characteristics.

Patient ID	Age at diagnosis	Sex	Prematurity, gestational week	Multifocal cutaneous IH
P1	8 mo	F	Y, 32 ^5^/_7_	Y
P2	3 mo	F	Y, 28 1/_7_	Y
P3	3 mo	F	N	Y
P4	2 mo	M	n/a	N
P5	2 mo	F	Y, 27 ^6^/_7_	Y
P6	2 mo	F	Y, 27 ^5^/_7_	Y
P7	5 days	F	N, 38 ^4^/_7_	Y
P8	2 mo	F	Y, 34 ^2^/_7_	Y
P9	6 mo	F	N, 38 ^2^/_7_	N
P10	4 mo	F	Y, 28 ^2^/_7_	Y
P11	at birth	F	Y, 36 ^5^/_7_	Y
P12	5 mo	F	Y, 25 ^4^/_7_	Y
P13	2 mo	M	n/a	n/a
P14	2 mo	F	Y, 28 ^5^/_7_	Y
P15	6 mo	F	Y, 28 ^1^/_7_	Y
P16	6 mo	M	Y, 27 ^2^/_7_	Y
P17	6 mo	F	Y, 30 ^0^/_7_	Y
P18	5 mo	F	n/a	n/a
P19	6 mo	F	Y, 26 ^0^/_7_	n/a
P20	3 mo	F	n/a	n/a
P21	5 mo	F	Y, 27 ^2^/_7_	Y
P22	4 mo	M	Y, 31 ^3^/_7_	n/a
P23	1 mo	M	N	Y
P24	6 mo	M	n/a	n/a

This table summarizes the demographic and clinical data for the patients involved in the study. mo, month, F, female, M, male, Y, yes, N, no, n/a, not available. The columns provide the age of the patient at the time of diagnosis, sex, prematurity and gestational age, and whether cutaneous hemangiomas are present. Additional relevant details, such as the presence of other conditions, are also included.

Nine patients (37.5%) were diagnosed between birth and two months, five (20.8%) between three and four months, nine (37.5%) between five and six months, and one (4.1%) between seven and eight months.

Gestational age was available for 19 patients: 15 were preterm (78.9%) and four at term (21%). Prematurity ranged from 25 ^4^/_7_ weeks (P12) to 36 ^5^­/_7_ weeks (P11).

Regarding cutaneous involvement, data was available for 18 patients. Among these, 16 patients (88.9%) had multifocal cutaneous IH, while P4 and P9 had no skin involvement (11.1%). P11 had a central nervous system IH (16).

### Laboratory findings

All laboratory findings are depicted in Table 2. Alpha-Fetoprotein (AFP) was within age-adjusted reference values in all tested patients (*n* = 14).

**Table 2:** Lab results at diagnosis.

Patient ID	AFP (ng/mL)	beta hCG (mIU/mL)	NSE (µg/l)	Thyroid hormones	proBNP (pg/mL)
P1	97,7	<0.3	22.3	n/a	n/a
P2	655	<0.25	17.4	TSH 6,75 µU/mL fT3 5,88 pmol/l fT4 14,8 pmol/l	**315**
P3	464	<0.25	23.6	TSH 2,39 µU/mL	**247**
P4	660	<0.25	n/a	TSH 3,17 µU/mL fT3 6,36 pmol/l fT4 17,5 pmol/l	**283**
P5	32,039	<0.3	31.9	TSH 3,10 µU/mL fT3 5,01 pmol/l fT4 17,4 pmol/l	**846**
P6	21,381	0,6	23.3	TSH 4,35 µU/mL fT3 4,90 pmol/l fT4 19,8 pmol/l	**1,766**
P7	16,580	<0.3	16.7	TSH 1,91 µU/mL fT3 6,50 pmol/l fT4 25,5 pmol/l	**2,329**
P8	308	<0.3	22.7	TSH 2,81 µIU/mL	**427**
P9	82,2	<0.3	25.5	**TSH 14,3 µIU/mL** fT3 4,14 pmol/l fT4 22,8 pmol/l	**410**
P10	418	<0.3	23.4	TSH 2,07 µIU/mL fT3 5,68 pmol/l fT4 17,2 pmol/l	122
P11	68,500	n/a	35.0	TSH 4,1 µIU/mL fT3 3,36 ng/dL **fT4 2,2 ng/dL**	n/a
P12	309	<0.3	**80.4**	TSH 5,84 µIU/mL fT3 7,3 pmol/l fT4 15,1 pmol/l	**235**
P13	858	<0.3	18.16	n/a	n/a
P14	n/a	n/a	n/a	n/a	n/a
P15	n/a	n/a	n/a	n/a	n/a
P16	n/a	n/a	n/a	n/a	n/a
P17	n/a	n/a	n/a	n/a	n/a
P18	n/a	n/a	n/a	n/a	n/a
P19	n/a	n/a	n/a	n/a	n/a
P20	n/a	n/a	n/a	n/a	**4,953**
P21	n/a	n/a	n/a	n/a	n/a
P22	n/a	n/a	n/a	n/a	n/a
P23	n/a	n/a	n/a	n/a	n/a
P24	190	<0.3	n/a	n/a	n/a

This table presents the lab results for each patient at the time of diagnosis. The reference values used for comparison are as follows: TSH 0.73- 8.35 µU/mL, fT3 3.30–8.95 pmol/l, and fT4 11.9–25.6 pmol/l. NSE <17 µg/l and Beta-HCG <5.3 mIU/mL. In neonates, reference values for proBNP are not well established. The reference value for AFP was based on Blohm et al. (33).

Neuron-Specific Enolase (NSE) was assessed in 12 patients. Two had normal values (<17 µg/L). Mild elevation (17.4–35 µg/L) was observed in 10 patients. P12 had a pathological level (80.4 µg/L), but urine catecholamines were normal.

Beta-hCG was measured in 13 patients, all within the normal range (<5.3 mIU/mL).

Thyroid parameters were analyzed in 11 patients, with nine showing unremarkable levels. In contrast, P9 with extensive liver involvement (>10 lesions, 40–45 mm in diameter) showed markedly elevated TSH (36.8 µU/mL, reference range 0.73–8.35 µU/mL), with normal free T3 (4.14 pmol/L), and T4 (22.8 pmol/L). P11, again with extensive liver involvement (>10 lesions, 12.8 mm in diameter), demonstrated slightly reduced free T4 levels at 2.2 ng/dL.

ProBNP levels were measured in 11 patients. It is important to note that in neonates, reference values for proBNP are not well established. Levels are typically elevated shortly after birth and decline physiologically within the first 1–3 months of life. Only P10 had a value <125 pg/mL. Others ranged from 235 pg/mL (P12) to 4,953 pg/mL (P20) (median 410 pg/mL. P6 and P7 also had elevated values (1,766 and 2,329 pg/mL) but normal echocardiography. P20, with diffuse IH and the highest proBNP (2329pg/mL), had an enlarged atrium and ventricle, impaired systolic function, and elevated right ventricular pressure. After propranolol, cardiac findings normalized (16).

### Imaging findings

All imaging findings are summarized in Table 3. This retrospective analysis included data from 24 patients who underwent imaging for liver lesion characterization. Among the 24 patients, 15 underwent only CEUS, 3 underwent only MRI, and 6 patients underwent both modalities. In total, 21 CEUS examinations and 9 MRI examinations, with 6 paired examinations were available for direct comparison.

**Table 3:** Lesion characteristics in CEUS and MRI.

Patient ID	max. diameter CEUS (mm)	max. diameter MRI (mm)	number of lesions CEUS	number of lesions MRI	RI A.hep.
P1	11–12	n/a	5–10	n/a	n/a
P2	8–9	n/a	>10	n/a	n/a
P3	13	10	>10	>10	n/a
P4	6–7	6	>10	>10	n/a
P5	10	3–5	5–10	5–10	n/a
P6	7	7	>10	>10	n/a
P7	n/a	8	n/a	5–10	n/a
P8	5	n/a	>10	n/a	n/a
P9	40–45	40–45	>10	>10	0.67
P10	n/a	9	n/a	5–10	n/a
P11	n/a	12,8	n/a	>10	n/a
P12	4	n/a	<5	n/a	n/a
P13	11	15	>10	>10	n/a
P14	8	n/a	>10	n/a	0.69
P15	6	n/a	>10	n/a	0.8
P16	20	n/a	>10	n/a	0.65
P17	9	n/a	5–10	n/a	n/a
P18	16	n/a	5–10	n/a	0.68
P19	10	n/a	>10	n/a	0.69
P20	30	n/a	>10	n/a	0.78
P21	14	n/a	>10	n/a	0.74
P22	8	n/a	>10	n/a	0.59
P23	18	n/a	>10	n/a	0.72
P24	19	n/a	>10	n/a	0.72

This table provides the maximum diameter and number of lesions observed in CEUS(CEUS) and magnetic resonance imaging (MRI) for each patient. P20 presented with diffuse liver involvement. Additionally, the Resistive index (RI) of the hepatic artery is listed, normal range 0.5–0.75 pathologic RI <0.5 und >0.8 (34). Bold values indicate results outside the established reference ranges.

Among the 21 patients examined with CEUS, maximum lesion diameters ranged from 4 to 45 mm. CEUS detected fewer than five lesions in one patient, five to ten lesions in four patients, and more than 10 lesions in 15 patients. In the nine patients who underwent MRI, three patients had 5–10 lesions, while six patients had more than 10 lesions. The six patients who underwent both imaging modalities demonstrated 100% concordance in lesion number category with <5, 5–10, or >10 lesions.

**Figure 2:**
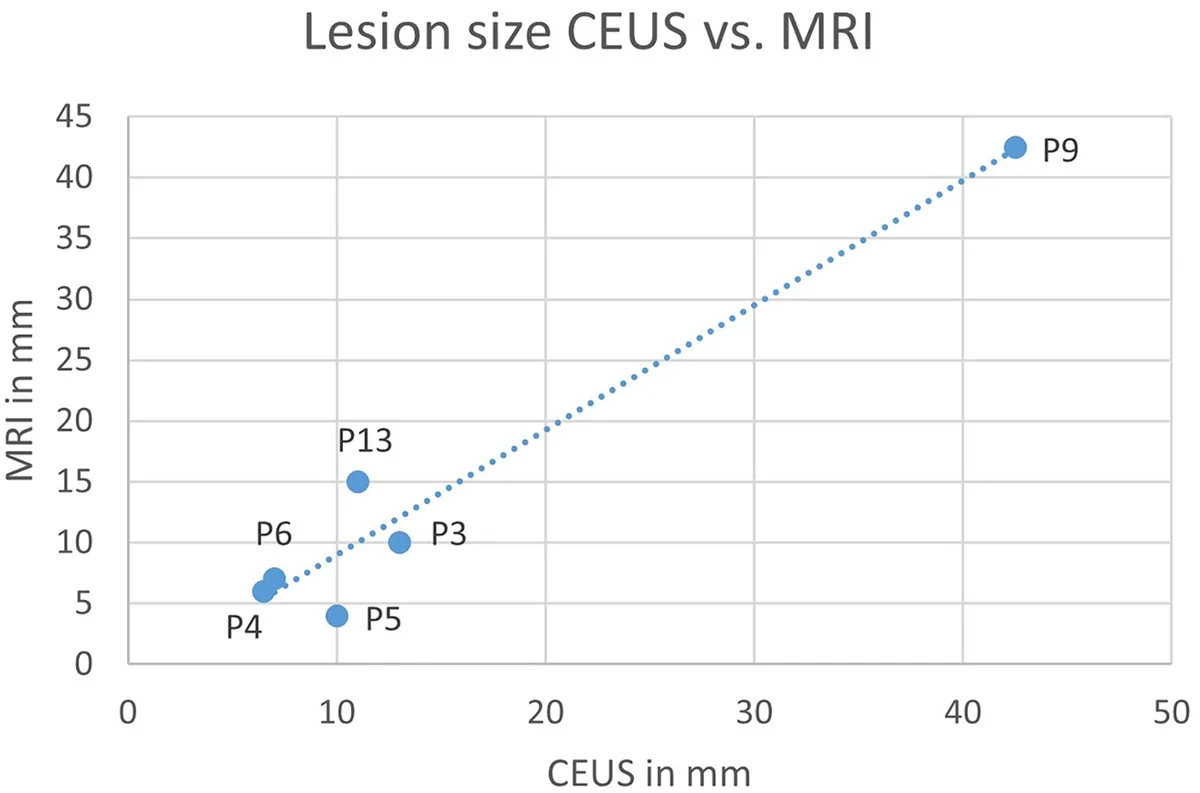
Scatter plot comparing the maximum lesion diameter (mm) measured by contrast-enhanced ultrasound (CEUS) and magnetic resonance imaging (MRI) in the six patients who underwent both imaging modalities. Each data point represents the maximum lesion diameter recorded for one patient as indicated by the patient number next to the data point. The dotted line illustrates the relationship between measurements obtained with the two imaging modalities. The exploratory Spearman rank correlation coefficient was *ρ* = 0.7714.

Due to perfect agreement, Cohen's Kappa was not calculable, but significant concordance can be assumed. Lesion size measurements revealed only minor variations of a few millimetres between modalities in three patients: P3 (CEUS 13 mm vs. MRI 10 mm), P5 (CEUS 10 mm vs. MRI 3–5 mm), and P13 (CEUS 11 mm vs. MRI 15 mm, respectively). The Spearman's rank correlation coefficient (*ρ*=0.7714) confirmed a strong positive correlation between modalities (Figure 2) (16).

### Treatment outcomes

Treatment outcomes are depicted in Table 4. Of 19 evaluable patients, 17 received propranolol as first-line therapy, six for 5–14 months, five ongoing at data collection, and six with incomplete data. Complete response to propranolol treatment was documented in eight patients. Two patients achieved complete regression under an observational approach, as did one patient for whom treatment information was unavailable. Four patients showed significant regression during treatment with residual lesion sizes of 3 and 4 mm (not documented in two patients, both of whom underwent therapy). P21 showed regression on CEUS, but treatment details were unavailable (16).

**Table 4:** Therapy and remission Status.

Patient ID	Therapy, duration	Outcome
P1	Propanolol, 5 mo	Regression (3 mm)
P2	Propanolol, 10 mo	Complete response
P3	Propanolol, 7 mo	Regression (4 mm)
P4	No therapy	Complete response
P5	Propanolol, 9 mo	Complete response
P6	Propanolol, duration unknown	Complete response
P7	Propanolol, 12 mo	Complete response
P8	Propanolol, ongoing	n/a
P9	Propanolol, ongoing	n/a
P10	Propanolol, ongoing	n/a
P11	Propanolol, ongoing	Regression
P12	Propanolol, ongoing	n/a
P13	Propanolol suggested	n/a
P14	Propanolol, duration unknown	Regression
P15	No therapy	Complete response
P16	n/a	n/a
P17	n/a	Complete response
P18	Propanolol, duration unknown	Complete response
P19	n/a	n/a
P20	Propanolol, 14 mo	Complete response
P21	n/a	Regression
P22	Propanolol, duration unknown	Complete response
P23	Propanolol, duration unknown	Complete response
P24	Propanolol, duration unknown	n/a

This table summarizes the treatment applied, duration of therapy, and the outcome for each patient. The outcome indicates whether a complete response or regression occurred.

## Discussion

Hepatic involvement is a rare manifestation of multifocal IH. In this study, we evaluated patient characteristics, laboratory parameters and imaging modalities in patients with hepatic IH to provide the basis for a non-invasive, fast and accurate diagnosis using CEUS.

Prematurity was highly prevalent in our cohort (79%), confirming its known association with multifocal IH (6). This highlights the importance of liver screening in preterm infants presenting with multiple cutaneous lesions.

Cardiac dysfunction occurred in only one infant with diffuse hepatic involvement, who presented with markedly elevated proBNP levels and abnormal echocardiographic findings. These abnormalities normalized after initiation of propranolol therapy, highlighting the reversibility of cardiac overload with treatment. ProBNP may therefore represent a sensitive marker of early cardiac strain in diffuse disease, although neonatal reference ranges remain uncertain (17, 18). These findings should be considered hypothesis-generating. In infants with extensive hepatic involvement or clinical signs suggestive of cardiac insufficiency, assessment of proBNP and echocardiography may be considered on an individual clinical basis.

Thyroid dysfunction, caused by type 3 iodothyronine deiodinase activity within hemangioma tissue, occurred in two patients with extensive liver involvement (19, 20). Newborn screening may miss this complication, as hypothyroidism typically develops later during tumor proliferation. Our findings thus support thyroid hormone testing in patients with hepatic IH (21).

Tumor markers AFP and beta-HCG were normal in all tested patients, while NSE levels were mildly elevated in 10/12 patients; this mild elevation was considered non-specific (possibly due to hemolysis). These findings should be considered descriptive. Tumor markers remain useful to evaluate malignant or ambiguous lesions (22, 23).

Our experience with CEUS suggests that it provides reliable imaging information. While visualization is limited to a specific liver window during examination, combining it with conventional ultrasound enables comprehensive assessment of both lesion number and diameter. Concordance with MRI in lesion number was 100%, and lesion size showed excellent concordance, to the extent that CEUS does not appear to be inferior to MRI in determining lesion size in our small cohort. Our observation aligns with studies reporting comparable detection of arterial hypervascularity of CEUS in adult liver lesions, due to real-time assessment (24). The high sensitivity of CEUS to vascular structures suggests that this technique allows accurate assessment of highly vascularized lesions such as hemangiomas and does not appear to be inferior to MRI in lesion size evaluation. This sensitivity to microvascular perfusion may explain the discrepant lesion size observed in patient P5 (CEUS 10 mm vs. MRI ∼3–5 mm), as adjacent lesions may have been interpreted as a single confluent lesion on CEUS. Overall, CEUS did not appear to underestimate lesion extent or severity compared with MRI in our cohort; however, the limited sample size precludes definitive conclusions. Recent studies have demonstrated CEUS' effectiveness in characterizing and stratifying focal liver lesions in children. These focused on various lesions, including hemangiomas, focal nodular hyperplasia, hepatocellular adenomas, and malignant tumors such as hepatoblastomas and metastases. Key findings indicate that early washout (≤45 s) strongly suggests malignancy, particularly hepatoblastoma in children under five. Hemangiomas are typically characterized by peripheral, discontinuous, globular hyperenhancement with centripetal fill-in. In very small lesions, however, this pattern may not be clearly identifiable; and lesions may present only as focal hyperenhancement compared with the surrounding liver parenchyma. This was observed in the smallest lesions of our cohort (4–5 mm). Overall, CEUS demonstrates high sensitivity (84.6–90.7%) and specificity (93.6%–100%) in differentiating benign from malignant lesions in the literature (13, 25–27).

In addition to diagnostic accuracy, CEUS offers significant advantages in pediatric liver imaging as it is safe, efficient, does not require sedation, and allows parental presence during the exam. Of note, sedation in our cohort could be avoided in patients undergoing CEUS while being administered in patients undergoing MRI, but the need for sedation during MRI may be avoided at other institutions, e.g., via the availability of feed-and-wrap techniques (12). The absence of CEUS-related adverse events in our cohort further supports the favorable safety profile previously described in the pediatric literature. The contrast agent is eliminated via the lungs, does not accumulate in the body and has no nephrotoxic effects (28). However. implementation of CEUS may face challenges, as it depends on local availability of appropriate ultrasound equipment, access to contrast agents, and personnel with specific expertise in pediatric CEUS (28–30). Operator dependence and the need for adequate training may limit reproducibility between centers. Nonetheless, based on our experience, CEUS may be considered a practical first-line approach in infants with multifocal cutaneous IH and multiple liver lesions, whereas MRI remains an important reference modality in ambiguous or complex cases, in order to avoid gadolinium exposure at this young age. Recent studies showed gadolinium accumulation in brain and other tissues, raising concerns about potential long-term toxicity, especially in the developing brains of children (31). Additionally, Gadolinium-based contrast agent have been associated with nephrogenic systemic fibrosis in patients including children with impaired renal function, although this risk has been significantly reduced with current guidelines (31, 32).

Because venous access is needed for CEUS, the recommended blood draw (e.g to check thyroid function) can be combined to reduce painful stimuli for affected infants. Measurement of additional laboratory parameters depends on the extent of hepatic lesions and likelihood of differential diagnoses and may include proBNP (e.g., in patients with clinical signs of cardiac insufficiency or with extensive liver lesions) and tumor markers (e.g., in patients with a lack of cutaneous hemangiomas or atypical ultrasound findings).

Follow-up imaging – usually performed with B-mode ultrasound – is essential to confirm regression, which may occur more slowly in hepatic than in cutaneous IH.

The retrospective design reflects real-world clinical decision-making, where the choice of imaging modality (CEUS vs. MRI) for further lesion characterization was individualized according to clinical characteristics.

Limitations nonetheless include the retrospective design, small sample size, and dependence on examiner expertise for CEUS interpretation. However, these constraints reflect the rarity of hepatic IH and the limited availability of large pediatric imaging datasets.

In conclusion, CEUS combined with conventional ultrasound appears to provide consistent information on hepatic IH, with high concordance to MRI in our small cohort. CEUS may offer added sensitivity for vascular structures. Its safety profile and feasibility in infants underline its potential value as part of the diagnostic workup. Accordingly, conventional ultrasound should be regarded as the first-line tool for screening and longitudinal follow-up of at-risk infants, while CEUS may serve as an additional modality for lesion characterization. MRI remains the gold standard, particularly in cases of diagnostic uncertainty or contraindications to CEUS. Future research should focus on prospective studies to validate these findings and establish standardized CEUS protocols.

## Data Availability

The datasets presented in this study can be found in online repositories. The names of the repository/repositories and accession number(s) can be found below: we have posted a preprint of this manuscript on medRxiv on Mai 10, 2025 (https://doi.org/10.1101/2025.05.08.25327084).

## Supplementary material

Supplementary Video 1 Contrast-enhanced ultrasound (CEUS) of an infantile hepatic hemangioma showing the typical enhancement pattern: early peripheral nodular enhancement with progressive centripetal fill-in during the arterial phase, followed by homogeneous enhancement in the later phases.
