# Supplementary material for: Evaluation of hepatic infantile hemangiomas: the potential of contrast-enhanced ultrasound
Source: Front Cardiovasc Med. 2026 Sep 8;13:1886544. doi: 10.3389/fcvm.2026.1886544 (PMC13593435; doi:10.3389/fcvm.2026.1886544)
Supplement: Supplementary file 1 [file Table1.docx]

|  | n |
| --- | --- |
| Sex |  |
| Female | 18/24 |
| Male | 6/24 |
| Gestational age (n.a. n=5) |  |
| Preterm | 15/19 |
| Term | 4/19 |
| Cutaneous involvement | 16/18 |

**Table 1S.** The table summarizes the demographic and clinical characteristics of the study population, including sex distribution, gestational age, and cutaneous involvement, along with their respective statistical significance (p-values).
